# Design of Transparent Metasurfaces Based on Asymmetric Nanostructures for Directional and Selective Absorption

**DOI:** 10.3390/ma13173751

**Published:** 2020-08-25

**Authors:** Dong Wu, Yang Meng, Chang Liu

**Affiliations:** 1 State Key Laboratory for Mesoscopic Physics, School of Physics, Peking University, Beijing 100871, China; 2 Department of Biomedical Engineering, School of Medicine, Tsinghua University, Beijing 100084, China; ericmeng007@tsinghua.edu.cn; 3 State Key Laboratory of Superlattices and Microstructures Institute of Semiconductors Chinese Academy of Sciences, Beijing 100083, China; liuchangmt@bupt.edu.cn

**Keywords:** absorber, metasurface, subwavelength structure, solar radiation, thermal management, directional absorption, metamaterial

## Abstract

Maximizing the solar heat gain through windows in winter and minimizing the solar radiation entering the room in summer are of great significance for the energy saving of buildings. Here, we present a new idea for transparent metasurfaces, based on asymmetric metal/insulator/metal (MIM) nanostructures, which can be switched back and forth between absorbing and reflecting solar radiation by reversing the sample orientation. Owing to the fundamental mode of a low-quality-factor resonance, a selective near-infrared absorption is obtained with an absorption peak value of 90% upon front illumination. The average solar absorption (45%) is about 10% higher than that (35%) of reported transparent absorbers. The near-infrared light is also strongly and selectively reflected upon back illumination and a reflection peak value above 70% is observed. Meanwhile, the average visible transmission of the metasurface is above 60%, which is about 1.6 times that (36%) of previous transparent metasurface absorbers. In addition, Cu material can replace the noble metals in this work, which will greatly reduce the manufacturing cost. Owing to the attractive properties of directional and selective absorption, passive operation mode, and low cost of the materials, the metasurfaces have promising prospects in building energy saving or other solar applications where surface transparency is desirable.

## 1. Introduction

As the energy consumption of buildings accounts for a great proportion of the social total energy consumption, it is imperative to open up new passive energy-saving technologies to improve the energy efficiency of buildings [1,2,3]. As an important part of building enclosures, the design of window systems is the most serious challenge for the energy saving of buildings and is also closely related to the degree of indoor comfort [2,3]. Windows with large areas in modern public buildings are required to provide good natural lighting but also to greatly increase solar heat gain. Solar heat gain is the main source of room cooling load, and the energy consumption of the air conditioning system is the main part of the energy consumption of the building’s operation in summer [3]. Therefore, how to partly hinder the solar radiation into the building is the key point of building energy efficiency work in summer [3]. In addition, to attain a comfortable indoor thermal environment, the energy consumed in the heating of buildings in winter accounts for a high proportion of building energy consumption [2]. In winter, because of low outside temperatures, the key factor of architecture energy saving is how to reduce the heat exchange of the architecture structure. Considering that windows are the most active positions of the architecture structure’s energy exchanging [2], the novel solar–thermal materials, converting solar energy into heat for raising its temperature, are promising and worth studying as a replacement for traditional glasses and a way to reduce the energy needed for home heating in winter [2,3], which is as important as shading solar heat in summer.

As a key technology for solar energy thermal utilization, many solar metasurface absorbers based on noble metals have been extensively investigated for applications [4,5,6,7,8,9,10,11,12] such as water desalination [6,13,14] and solar thermophotovoltaic [15]. Most of these traditional metasurface absorbers generally adopt an opaque metal substrate to eliminate the transmission of all light [4,7,16,17,18,19,20,21,22,23,24,25,26,27,28,29,30,31], but these are not suitable for the requirements of windows or other applications with a transparent surface [1,2,4,12,32,33]. In recent years, due to their great potential value in environmental protection and sustainable energy development, transparent solar technology has attracted increasing attention [1,2,12,33,34]. For example, in 2017, Gustav et al. proposed and demonstrated a transparent solar absorber built on sparse arrays of multielement gold plasmonic nanoantennae [2], which can be implemented on regular window glass. Transparent solar absorbers have a high transmittance in the visible region and can raise their temperature by up to 8 K upon solar irradiation [2]. These properties allow the surface of architectural glass to be turned into solar harvesting arrays without impacting the function of daylighting [2]. In 2018, Efstratios et al. showed a transparent plasmonic metasurface consisting of closely packed Au particles [12], which can convert absorbed solar energy into heat. The transparent metasurfaces had a mean absorption value of 37% and a mean transmittance value of 36% in the visible region [12]. The basic idea of this solar–thermal technology is to balance the transparency and visible light absorption of plasmonic metasurfaces. Therefore, using this strategy, higher transparency means lower visible light absorption and vice versa, which indicates that the metasurfaces based on such an idea cannot simultaneously achieve a high transparency and a good solar–thermal response. In 2019, Christopher et al. demonstrated a novel approach by employing transparent plasmonic metasurfaces harnessing the broadband spectrum of the sun [33] to efficiently heat up a surface. The transparent metasurface absorber consisted of a metal−dielectric nanocomposite of gold nanoparticles embedded in titania on glass substrates. The visible light transmission (400−750 nm) of the metasurface reached 36%. However, because of its good solar absorption performance, for practical application in the windows of buildings, these previously reported transparent solar absorbers are energy-saving in winter but energy-wasting in summer. In addition, the large-scale and further deployment of these reported transparent solar absorbers based on metasurfaces are also limited by the cost of noble metals, such as gold and silver [5,6,8,9,10,12,28,35,36,37]. Thus, it is a highly challenging and meaningful task to design and realize a metasurface using inexpensive materials, which can simultaneously achieve visible transparency, selective near-infrared absorption for front illumination, and selective near-infrared reflection for back illumination.

For the purpose of building energy saving in both winter and summer, three characteristics of the metasurface absorber are proposed and summarized as follows: (1) the structure should have a high transmission in the visible region; (2) the structure should have a high near-infrared absorption for front illumination; (3) the structure should strongly reflect the incident near-infrared light for back illumination. Such transparent metasurfaces can be used to absorb or reflect solar radiation in different seasons by reversing the window orientation. Here, we present and numerically demonstrate a new idea for a transparent solar absorber based on asymmetric metasurfaces. The design of an asymmetrical structure makes the front and backside of the metasurfaces absorb or reflect near-infrared light differently. Owing to the fundamental mode of a low-quality-factor resonance, the proposed metasurface strongly and selectively absorbs near-infrared light and exhibits an absorption peak above 90% at 913.5 nm for front illumination. The solar absorption performance of the proposed metasurface is much larger than that of previously reported transparent solar absorbers based on metasurfaces [2,12,33]. Meanwhile, the near-infrared incident light is strongly reflected for back illumination and a reflection peak above 70% is observed around 913 nm. Meanwhile, the average transmission of the metasurface in the visible range (400–750 nm) is above 60%, which is about 1.6 times that (36%) of previous transparent metasurface absorbers [12,33]. In addition to the noble metal, other non-noble metals, such as copper, can also be used as a reflective layer for back illumination, which is important for reducing the manufacturing cost in their practical application.

## 2. Materials and Methods

**Figure 1:**
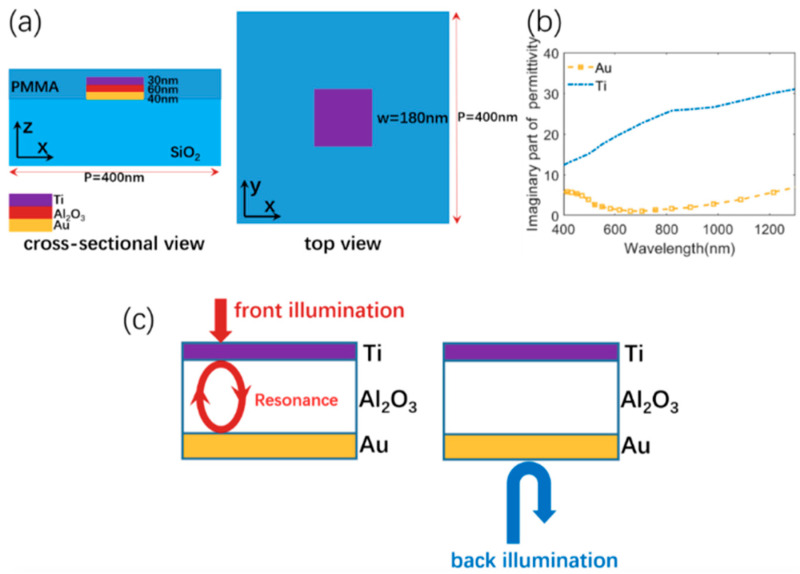
(**a**) Design of the visible-transparent metasurface absorber. (**b**) Imaginary parts of the permittivities for Ti and Au. (**c**) Physical mechanism for the selective near-infrared absorption for front illumination and selective near-infrared reflection for back illumination.

We proposed a metasurface absorber consisting of periodic metal/insulator/metal (MIM) units located on a SiO_2_ substrate, maintaining a large distance between these MIM units to ensure high transmission in the visible region, as shown in Figure 1a. Then, considering the different requirements of the optical properties in the near-infrared region for front and back illumination, the MIM structure was designed using a unique asymmetric design along the incident wave direction, which had two metal layers with different metal materials. As shown in Figure 1b, Ti was chosen as the upper metal layer because of its higher optical loss (or imaginary part of the effective permittivity), which is beneficial to near-infrared absorption upon the front illumination. The incident light can also be reflected back and forth between the two dielectric-metal interfaces in the MIM structure, constructing a resonator, as shown in Figure 1c. The dielectric (Al_2_O_3_) layer is designed to provide phase adjusting capability to the resonator. Owing to a more negative real part of dielectric permittivity ε of Au in the near-infrared region, the Au film was used as a reflective layer for the near-infrared light from the back side. A lossless PMMA (polymethyl methacrylate) layer with a thickness of 150 nm was set to be an antireflection coating, to decrease the impedance mismatch between the designed structure and the air. Then, the finite-difference time domain (FDTD) method was performed to calculate the optical properties of the proposed structure. A normally incident, transverse magnetic(TM)-polarized light was incident along the negative z direction with the polarization along the x direction. Periodic boundary conditions were applied in the x and y directions. Perfectly matched layers (PML) were implemented at the upper and bottom boundary of the model. The refractive indexes of Ti, Au, and Al_2_O_3_ were taken from experimental data [38]. In this simulation, we set the structural parameters as: P = 400 nm, w = 180 nm, t_Ti_ = 30 nm, t_Al2O3_ = 60 nm, and t_Au_ = 40 nm. The absorption A can be defined as A = 1 − R (reflection) − T (transmission).

## 3. Results and Discussion

**Figure 2:**
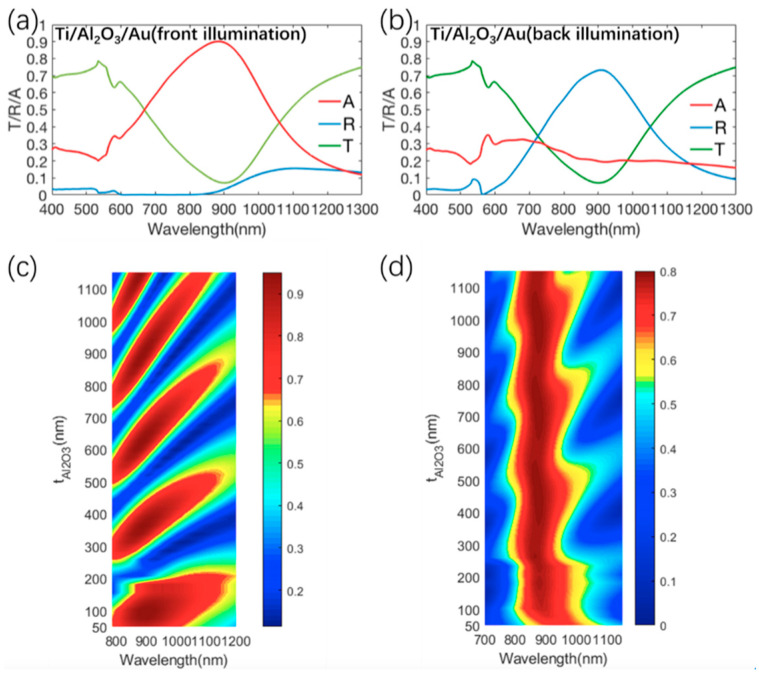
(**a**) Simulation absorption/transmission/reflection spectra of the metasurface consisting of Ti/Al_2_O_3_/Au structure for front illumination (**b**) Simulation absorption/transmission/reflection spectra of the metasurface consisting of Ti/Al_2_O_3_/Au structure for back illumination. (**c**) Simulated absorption spectra of the metasurface consisting of Ti/Al_2_O_3_/Au with various t_Al2O3_ for front illumination. (**d**) Simulated reflection spectra of the metasurface consisting of Ti/Al_2_O_3_/Au with various t_Al2O3_ for back illumination.

As shown in Figure 2, the absorption/transmission/reflection spectra of the metasurface composed of Ti/Al_2_O_3_/Au are calculated in the visible and near-infrared region, which was illuminated from the front side or back side. Owing to the small area ratio of metal layer in a unit cell, the metasurface was largely transparent (with an average transmission above 60%) in the visible region (400–760 nm). Interestingly, we also found that the metasurface had a strong directionality in near-infrared absorption. For front illumination, the proposed metasurface strongly absorbed near-infrared light and exhibited an absorption peak above 90% at 890 nm, as shown in Figure 2a. Meanwhile, the near-infrared incident light was strongly reflected for back illumination and a reflection peak above 70% was clearly observed around 910 nm, as shown in Figure 2c. In addition, other similar transparent dielectrics, such as SiO_2_ or TiO_2_ polymers, can also be used as the dielectric layers. For example, the optical properties of the metasurface, comprised of Ti/SiO_2_/Au, were calculated and are depicted in Figure S1. The structures comprised of Ti/SiO_2_/Au had similar optical responses to the metasurface consisting of the Ti/Al_2_O_3_/Au structure shown in Figure 2a,b. These simulation results show that our proposed transparent absorber can efficiently absorb the solar energy for front illumination, while it keeps strong near-infrared reflection by reversing the sample orientation.

**Figure 3:**
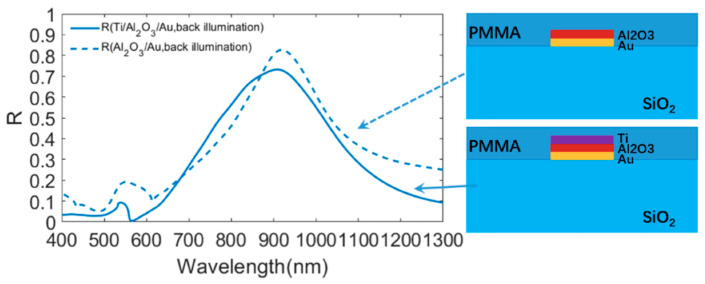
Comparisons of reflection spectra between the metasurface consisting of Ti/Al_2_O_3_/Au and the metasurface consisting of Al_2_O_3_/Au upon back illumination.

**Figure 4:**
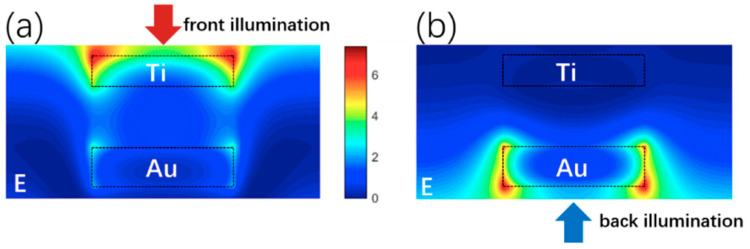
(**a**) Distributions of the electric field E at the resonant wavelength of 890 nm. (**b**) Distributions of the electric field E at the resonant wavelength of 910 nm.

Next, to prove that the structure operated as a resonator upon front illumination, the absorption spectra of the metasurface upon front illumination were studied by varying the thickness (t_Al2O3_) of the dielectric (Al_2_O_3_) layer, as shown in Figure 2c. According to Figure 2c, with the increase in the thickness of the Al_2_O_3_ layer, it can be observed that the number of resonance modes in the MIM structure gradually increased upon front illumination. This indicates that the MIM structure in Figure 1a operated as a resonator, which is consistent with the design of the metasurface in Figure 1c. Meanwhile, we also found that the lower resonance mode—corresponding to the smaller thickness of the dielectric layer—had a larger bandwidth. The thickness t_Al2O3_ = 60 nm was chosen to obtain the fundamental mode with a larger bandwidth of the absorption peak, which is beneficial for harvesting solar energy. Therefore, according to the desired absorption band, the absorption band can be controlled by adjusting the thickness of the insulator layer. In addition, the resonance mode of the asymmetric Fabry–Perot cavity shaped by this MIM structure follows the equation of the form [39].

(1) $$2\left(\frac{2\pi }{\lambda_{res}}\right)n_{i}t_{i}+\emptyset_{b}+\emptyset_{t}=2\pi m$$

where *λ_res_* is the resonance wavelength, *n_i_* and *t_i_* are the refractive index and thickness of the insulator layer, respectively, and m is an integer number which determines the order of cavity mode. $\emptyset_{b}$ and $\emptyset_{t}$ are the phase shift acquired from the reflection from the bottom and top metal layers, respectively. It can be seen that, by increasing *t_i_*, the resonance wavelength *λ_res_* will red-shift, which is consistent with the results in Figure 2c. In addition, with the increase in the thickness (*t_i_*) of the insulator layer, it can also be observed that the number of resonance modes will increase, which is also consistent with the results in Figure 2c. Then, the reflection spectra of the metasurface upon back illumination were also studied by varying the thickness of the Al_2_O_3_ layer, as shown in Figure 2d. It was observed that the wavelength position of the reflection peak did not change with the variation in the thickness of the Al_2_O_3_ layer. The comparisons of reflection spectra between the metasurface consisting of Ti/Al_2_O_3_/Au and the metasurface consisting of Al_2_O_3_/Au upon back illumination were calculated and are depicted in Figure 3. Clearly, the strong near-infrared reflection upon back illumination is independent from the MIM structure and is closely associated with the optical properties of the gold layer. Thus, the strong near-infrared reflection upon back illumination is caused by the Au layer, which is consistent with the design of the metasurface in Figure 1c. Moreover, the distributions of the electric field E at these two resonant wavelengths for front and back illumination were also calculated and are depicted in Figure 4a,c, respectively. It can be clearly observed that the electric field for front illumination was enhanced and localized at the edge of the Ti layer and the electric field for back illumination was localized at the edge of the gold layer. These results show that the larger optical loss of Ti is beneficial for constructing a low-quality-factor (high-loss) resonator to obtain a wider near-infrared absorption upon front illumination, and the strong near-infrared reflection upon back illumination is closely associated with the optical properties of the gold layer. For comparison, the absorption/reflection properties of the periodic Au/Al_2_O_3_/Au structures are studied and depicted in Figure S2. It can be seen that this symmetric metasurface, based on the symmetrical structure, reflects incident light broadly across the near-infrared spectrum for both front and back illumination. The absorption/reflection of this metasurface only has a slight difference for front and back illumination, which is related to the difference in the refractive index of the substrate and the surrounding air. Based on these differences between the two metasurfaces consisting of Ti/Al_2_O_3_/Au and Au/Al_2_O_3_/Au, it is easy to see that the strong directional absorption of our transparent metasurface is closely associated with the asymmetric design along the incident wave direction.

**Figure 5:**
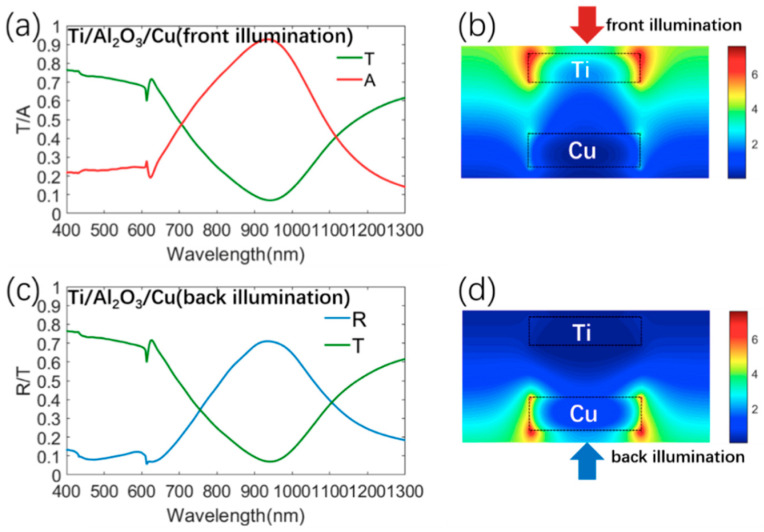
(**a**) Simulation absorption/transmission spectra of the metasurface consisting of Ti/Al_2_O_3_/Cu structure for front illumination, and (**b**) distributions of the electric field E at the resonant wavelength of 913.5 nm. (**c**) Simulation reflection/transmission spectra of the metasurface consisting of Ti/Al_2_O_3_/Cu structure for back illumination, and (**d**) distributions of the electric field E at the resonant wavelength of 913 nm.

**Figure 6:**
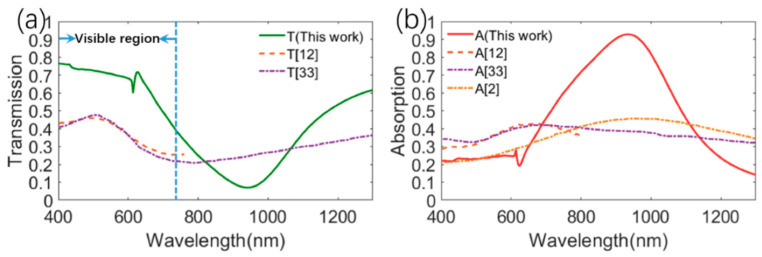
Comparisons of (**a**) transmission and (**b**) absorption performances between our metasurface absorbers and other reported transparent absorbers [2,12,33].

As discussed before, the strong directional absorption/reflection can be achieved by the metasurfaces comprised of Ti/Al_2_O_3_/Au structures. However, the high cost of the Au material (about USD 64,845 per kilogram) will limit the further development and application of the proposed transparent absorber. Then, we found that single Cu (about USD 576.4 per kilogram) film also possesses high reflection in the near-infrared region, as shown in Figure S3. Meanwhile, Cu and Au have a similar imaginary part of the dielectric constant in the visible–near infrared(NIR) region, as shown in Figure S4. Then, Cu material was used to replace the Au material in our structure in the following sections. The optical properties of the metasurface comprised of Ti/Al_2_O_3_/Cu were calculated and are depicted in Figure 5. The average visible transmission value in the visible region was above 60%, which is about 1.6 times that (36%) of previous transparent metasurface absorbers [12,33]. For front illumination, the proposed metasurface strongly and selectively absorbed near-infrared light and exhibited an absorption peak above 90% at 913.5 nm. Meanwhile, the near-infrared incident light was strongly reflected for back illumination and a reflection peak above 70% was observed around 913 nm. Then, the distributions of the electric field E at the resonant wavelengths were also calculated and are depicted in Figure 5b,d, respectively. The electric field for front illumination was localized at the edge of the Ti layer and the electric field for back illumination was localized at the edge of the Cu layer. Clearly, the absorption/transmission/reflection spectra of the metasurface using Cu are similar to the metasurface using Au, and thus it is more economic to realize the desirable optical properties using the metasurfaces with inexpensive materials. Moreover, Figure 6 compares the transmission and absorption characteristics of our metasurface with those of the state-of-the-art transparent solar absorbers [2,12,33]. The average visible transmission value in the visible region was above 60%, which is about 1.6 times that (36%) of previous transparent metasurface absorbers [12,33]. The average solar absorption (45%) of our transparent absorber is about 10% higher than that (35%) of reported transparent absorbers [12,33]. Clearly, our metasurface has much better visible transparency and higher solar absorption than previously reported transparent solar absorbers [2,12,33].

**Figure 7:**
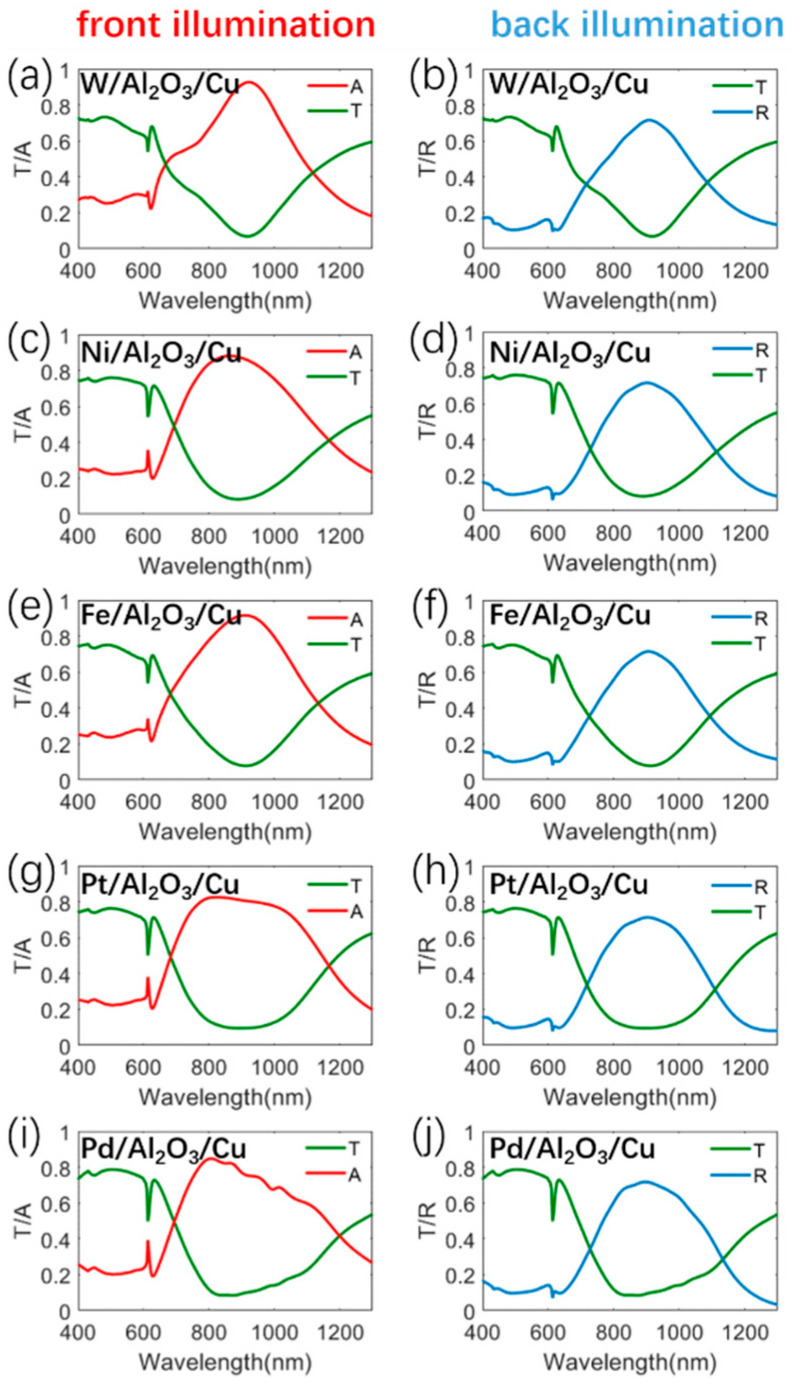
Simulation optical responses of the metasurface consisting of W/Al_2_O_3_/Cu structures for (**a**) front illumination and (**b**) back illumination. Simulation optical responses of the metasurface consisting of Ni/Al_2_O_3_/Cu structures for (**c**) front illumination and (**d**) back illumination. Simulation optical responses of the metasurface consisting of Fe/Al_2_O_3_/Cu structures for (**e**) front illumination and (**f**) back illumination. Simulation optical responses of the metasurface consisting of Pt/Al_2_O_3_/Cu structures for (**g**) front illumination and (**h**) back illumination. Simulation optical responses of the metasurface consisting of Pd/Al_2_O_3_/Cu structures for (**i**) front illumination and (**j**) back illumination.

In addition, the absorption metal for front illumination is also not limited to Ti. In fact, the metal materials, such as W, Ni, Fe, Pt, or Pd, also have larger imaginary parts of the permittivities, which are depicted in Figure S4. These materials can probably be utilized as an absorptive metal layer of the metasurface absorber for front illumination, owing to their higher optical loss. To prove the validity of our analysis, we give a detailed calculation for the metasurfaces based on these alternatives, as shown in Figure 7. As expected, the structures with these alternatives have similar optical characteristics to the metasurface consisting of the Ti/Al_2_O_3_/Cu structure shown in Figure 2a,b. These results show that these structures are highly transparent to visible light. At the same time, for the metasurfaces with the absorptive layer using W, Ni, Fe, Pt, or Pd, the near-infrared absorption values at the resonance wavelengths reach as high as 92.7%, 88.1%, 91.5%, 82.5%, and 85%, respectively. The absorption performance of these metasurfaces is much better than that of previously reported transparent solar absorbers [2,12,33]. For back illumination, these structures can also strongly reflect the near-infrared light. The demonstration in this section enables us to prove the validity of the asymmetric structure model based on high-loss metal/insulator/low-loss metal with a large variety of low-cost materials, and this may be helpful to further studies and applications.

**Figure 8:**
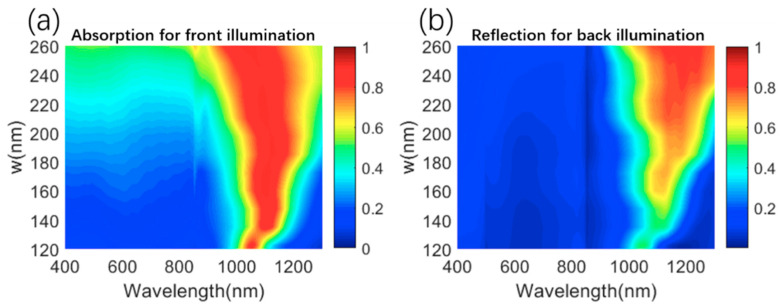
(**a**) Simulated absorption spectra of the metasurface composed of Ti/Al_2_O_3_/Cu with various w for front illumination. (**b**) Simulated absorption spectra of the metasurface composed of Ti/Al_2_O_3_/Cu with various w for back illumination.

To further investigate the influence of geometric structure on optical properties, the absorption/reflection/transmission spectra of the metasurface are studied by varying the side length w of the MIM structure with fixing the thickness of each layer, as shown, respectively, in Figure 8a,b and Figure S5. Clearly, with the increase in w, the near-infrared absorption for front illumination and the reflection in the near-infrared region for back illumination gradually redshift and is enhanced. Meanwhile, the visible transmission is maintained above 50% within a wide range of w (120–200 nm). The enhancement of the transmission with decreasing w can be easily explained by the smaller area ratio of the metal layer. Thus, the absorption/reflection/transmission properties of our proposed structure can be adjusted within a large range to satisfy different requirements for practical applications with transparent surfaces. In addition, the influence of changing the thickness of the metal layer and the periodicity P is also calculated and analyzed in Figures S6–S9. Moreover, the polarization independence is an important characteristic of the solar absorbers because the solar radiation is randomly polarized. As shown in Figure S10, the influence of the polarization angle on the optical response of the designed structure is studied for front and back illumination, respectively. Clearly, for a specific wavelength, the optical response does not change when the polarization angles change from 0° to 90°, which indicates its independence from the polarization of the incident light. The reason for polarization independence is that, for a specific polarization angle between 0° and 90°, the electric field can be decomposed into transverse electric (TE) and TM polarization lights, and meanwhile the optical response of the metasurface for the TM and TE polarization configuration is the same, owing to the high symmetry of the designed structure in the direction perpendicular to the incident light. From the above analyses, the optical responses of the proposed metasurface are insensitive to the polarization angle.

## 4. Conclusions

In this work, we proposed and demonstrated a transparent solar metasurface with directional and selective near-infrared light absorption. The simulation results show that the average transmission of the metasurface in the visible range (400–760 nm) was above 60%, which is about 1.6 times that (36%) of previous transparent metasurface absorbers [12,33]. For front illumination, the proposed metasurface strongly absorbed near-infrared light and exhibited an absorption peak above 90% at about 931.5 nm. The solar absorption performance of our transparent metasurface is much larger than that of previously reported transparent solar metasurface absorbers. Meanwhile, the near-infrared incident light was strongly reflected upon back illumination and a reflection peak above 70% was observed around 931 nm. Such directional absorption of solar light is attributed to the design of the asymmetric metasurfaces comprised of a high-loss metal film and a low-loss metal film spaced by a dielectric layer. The top metal layer, with higher optical loss, is responsible for generating strong near-infrared absorption for front illumination. The bottom metal layers, with lower optical loss, are used to enhance the reflectivity in the near-infrared wavelength range for back illumination. In addition, we can apply non-noble, low-loss metals, such as copper, instead of the noble metals often used in traditional metasurfaces, which can greatly reduce the manufacturing cost. This study has demonstrated the broad class of transparent metasurfaces that can be used to absorb or reflect solar radiation by reversing the sample orientation, which may have promising prospects in building energy saving or other solar applications where surface transparency is desirable.

## Supplementary Materials

The following are available online at https://www.mdpi.com/1996-1944/13/17/3751/s1, Figure S1: Optical responses of the metasurface consisting of Ti/SiO_2_/Au structure. Figure S2: Optical responses of the metasurface consisting of Au/Al_2_O_3_/Au structure. Figure S3: Simulation absorption/reflection spectra of the 100nm Ti, Au and Cu film. Figure S4: Imaginary parts of the permittivities for Cu, Au, Ti, Pt, W, Ni, Fe and Pd. Figure S5: Simulated absorption spectra of the metasurface composed of Ti/Al_2_O_3_/Cu with various w. Figure S6: Optical responses of the metasurface consisting of Ti/Al_2_O_3_/Cu with various h_Au_. Figure S7: Optical responses of the metasurface consisting of Ti/Al_2_O_3_/Cu with various h_Ti_. Figure S8: Optical responses of the metasurface consisting of Ti/Al_2_O_3_/Cu with various P. Figure S9: Optical responses of the metasurface composed of only Au layer with various P. Figure S10: Optical responses of the metasurface consisting of Ti/Al_2_O_3_/Cu with various polarization angle.
