# Supplementary material for: Design of Transparent Metasurfaces Based on Asymmetric Nanostructures for Directional and Selective Absorption
Source: Materials (Basel). 2020 Aug 25;13(17):3751. doi: 10.3390/ma13173751 (PMC7504014; doi:10.3390/ma13173751)
Supplement: Supplementary file 1 [file materials-13-03751-s001.pdf]

materials

MDPI

Article

Design of Transparent Metasurfaces Based on Asymmetric Nanostructures for Directional and Selective Absorption

Dong Wu [1,†,*], Yang Meng [2,†] and Chang Liu [3]

[1] State Key Laboratory for Mesoscopic Physics, School of Physics, Peking University, Beijing, 100871, China; dong_wu@pku.edu.cn
[2] Department of Biomedical Engineering, School of Medicine, Tsinghua University, Beijing, 100084, China; ericmeng007@tsinghua.edu.cn
[3] State Key Laboratory of Superlattices and Microstructures Institute of Semiconductors, Chinese Academy of Sciences, Beijing, 100083, China; liuchangmt@bupt.edu.cn
* Correspondence: dong_wu@pku.edu.cn (D.W.)
† These authors contributed equally to this work.

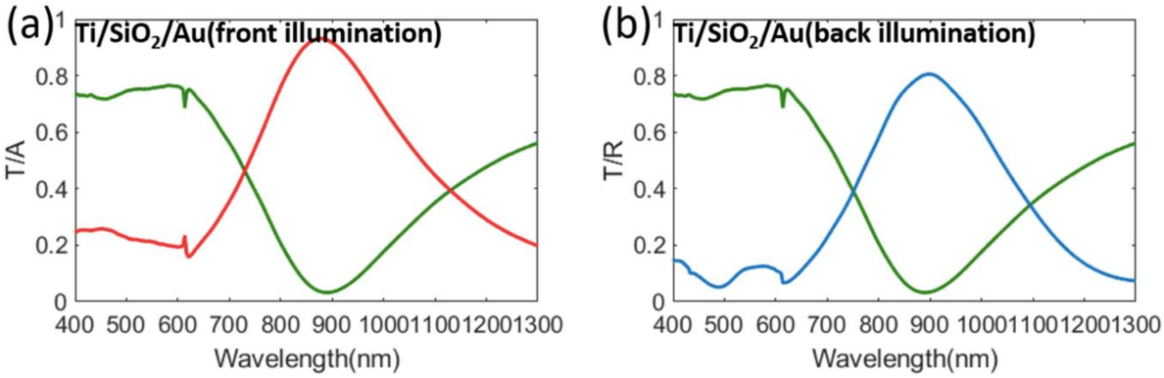

Figure S1. Optical responses of the metasurface consisting of Ti/SiO_2/Au structure. (**a**) Simulation absorption/transmission spectra of the metasurface consisting of Ti/SiO_2/Au structure for front illumination, and (**b**) Simulation reflection/transmission spectra of the metasurface consisting of Ti/SiO_2/Au structure for back illumination.

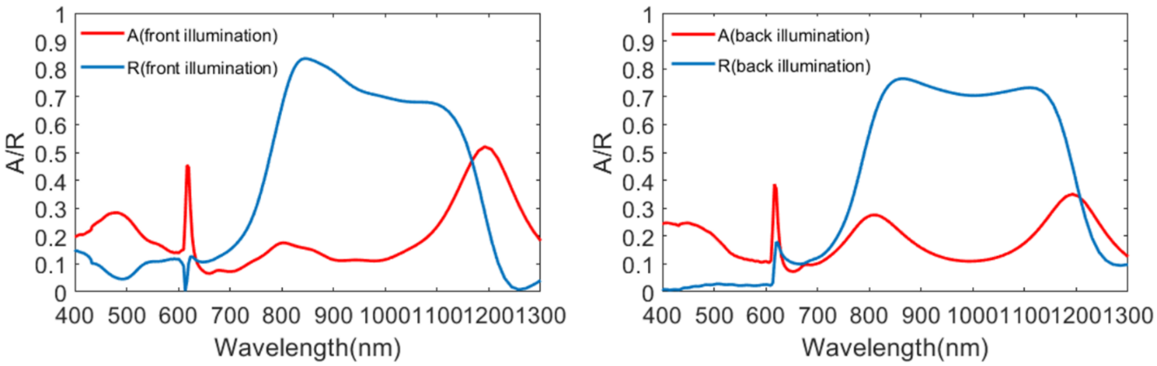

Figure S2. Optical responses of the metasurface consisting of Au/Al_2O_3/Au structure. Simulation absorption/reflection spectra of the metasurface consisting of Au/Al_2O_3/Au structure for (**a**) front and (**b**) back illumination.

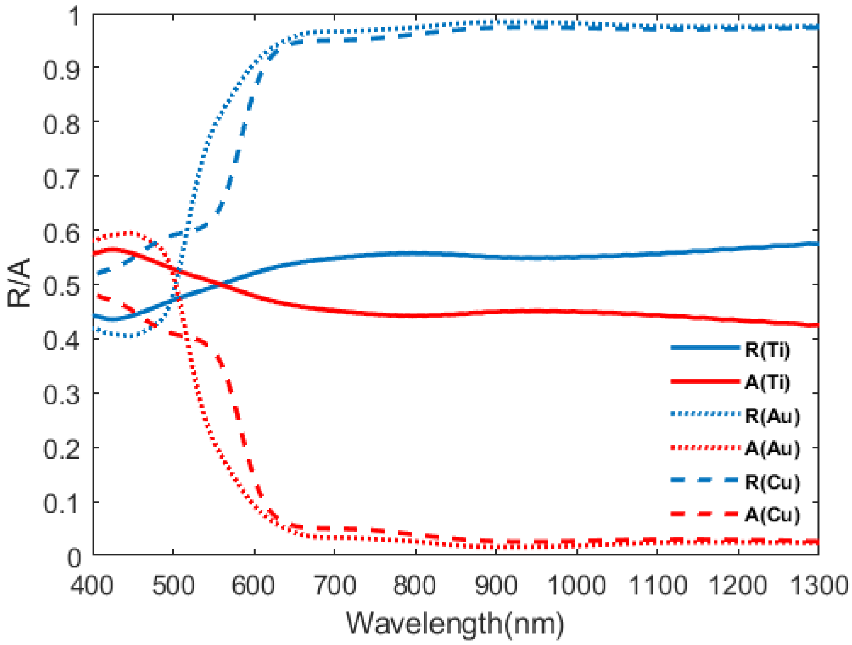

Figure S3. Simulation absorption/reflection spectra of the 100nm Ti, Au and Cu film, respectively.

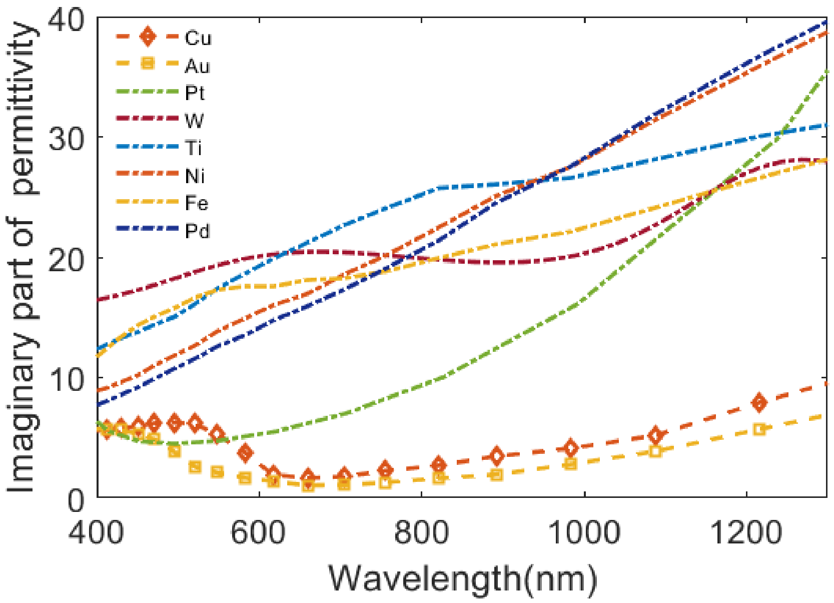

Figure S4. Imaginary parts of the permittivities for Cu, Au, Ti, Pt, W, Ni, Fe and Pd.

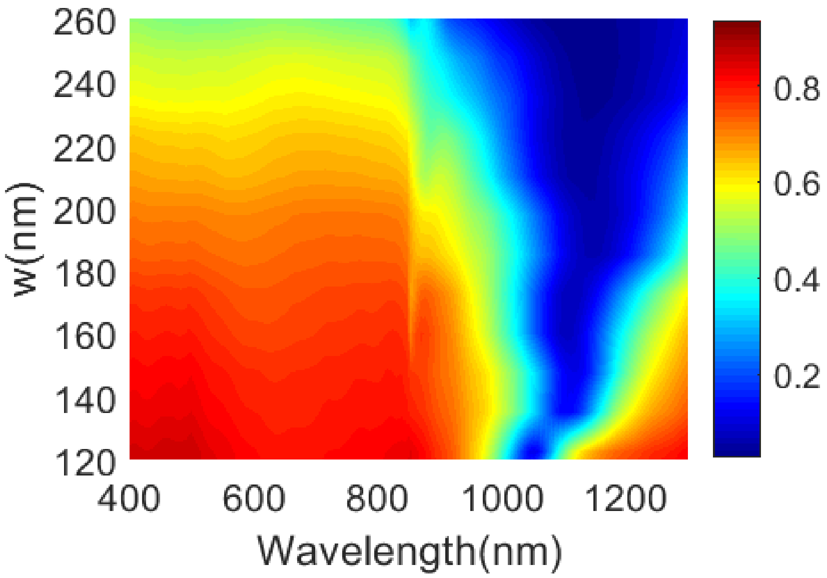

Figure S5. Simulated absorption spectra of the metasurface composed of Ti/Al_2O_3/Cu with various w.

As shown in Figure S6 and Figure S7, the transmission/absorption spectra of the metasurface are studied by varying the thickness (h_{Ti}, h_{Au}) of the metal Ti and Au, respectively. Clearly, according to Figure S6 and Figure S7, the selective visible transmission and selective infrared absorption are well maintained in a wide range of h_{Ti} and h_{Au}. The visible transmission is slightly enhanced and the infrared absorption slightly decreases with the increase of h_{Au}. Besides, the visible is slightly enhanced and the bandwidth of the infrared absorption gradually becomes narrower with the increase of h_{Ti}. Particularly, by the selection of h_{Ti}=30nm and h_{Au}=40nm, a high transmission is obtained for the wavelength region less than 780nm and meanwhile a selective absorption is obtained for the wavelength region beyond 780nm.

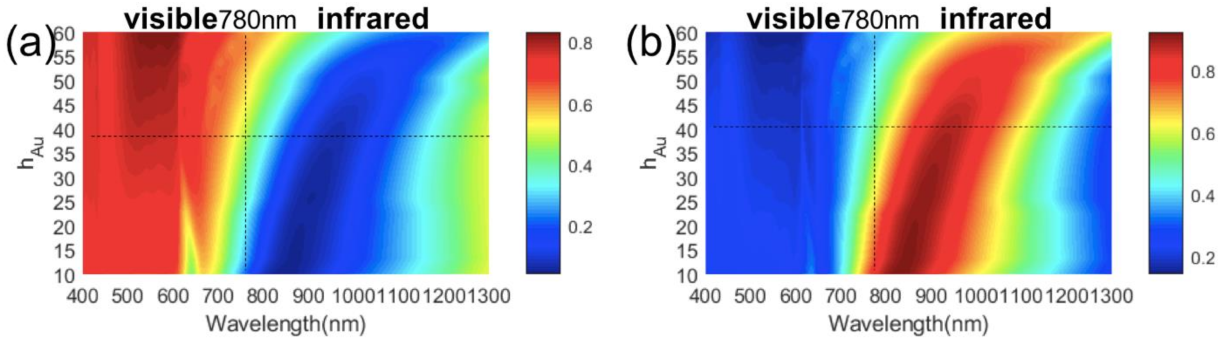

Figure S6. Optical responses of the metasurface consisting of Ti/Al_2O_3/Cu with various h_{Au}. Simulated (**a**) transmission and (**b**) absorption spectra of the metasurface composed of Ti/Al_2O_3/Cu with various h_{Au} for front illumination.

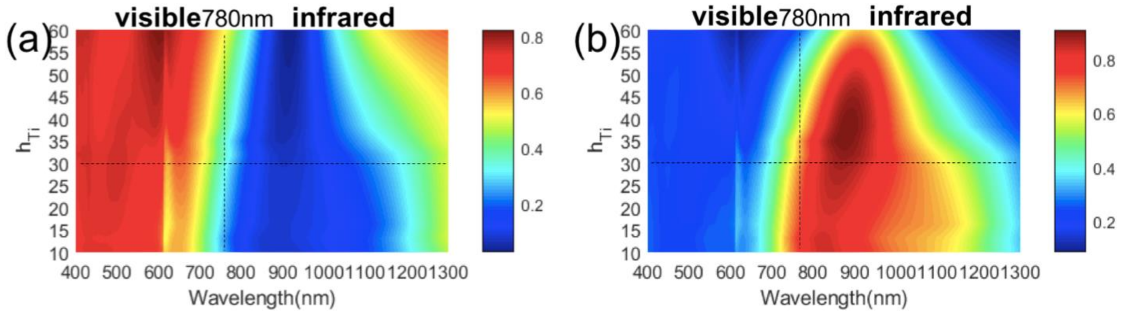

Figure S7. Optical responses of the metasurface consisting of $Ti/Al_2O_3/Cu$ with various h_{Ti}. Simulated (**a**) transmission and (**b**) absorption spectra of the metasurface composed of $Ti/Al_2O_3/Cu$ with various h_{Ti} for front illumination.

As shown in Figure S8, the transmission/absorption spectra of the metasurface are studied by varying the periodicity P, respectively. With the increase of periodicity P, the transmission of the metasurface is obviously enhanced, which can be easily explained by the smaller area ratio of metal layer. Meanwhile, the infrared absorption obviously decreases and red-shift when periodicity P increase. For periodicity P=400nm, a high transmission is obtained for the wavelength region less than 780nm and meanwhile a selective absorption is obtained for the wavelength region beyond 780nm. Then, in Figure S9, we also calculate the absorption spectra of the structure consisting of only Au layer (without the insulator and Ti layers). The absorption in Figure S9 obviously red-shift and slightly increases when the periodicity increases. The absorption in Figure S9 can be contributed to the resonance of Au layer and is relatively weak when periodicity is 400nm. Thus, for the original MIM structure with periodicity of 400nm, the optical response is mainly contributed to the cavity resonance of MIM structure(according to Figure 2c), and the resonance of Au layer also plays a relatively little role in the absorption enhancement. Moreover, for the original MIM structure with periodicity larger than 600nm, the absorption becomes narrow and is mainly contributed the resonance of Au layer.

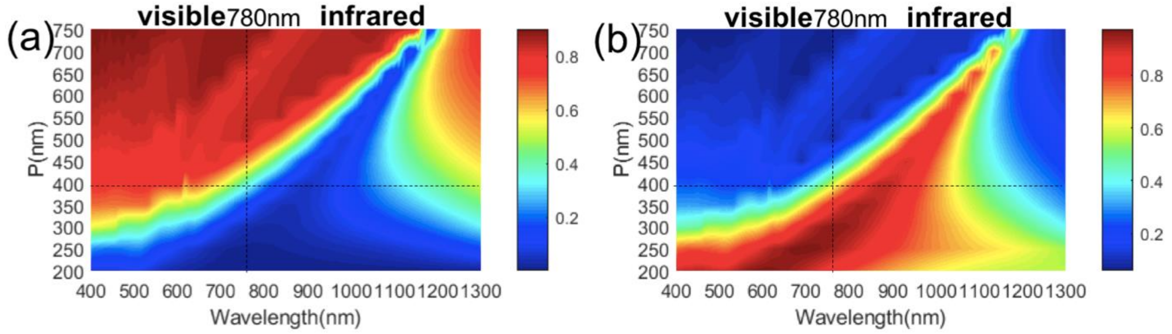

Figure S8. Optical responses of the metasurface consisting of $Ti/Al_2O_3/Cu$ with various P. Simulated (**a**) transmission and (**b**) absorption spectra of the metasurface composed of $Ti/Al_2O_3/Cu$ with various P for front illumination.

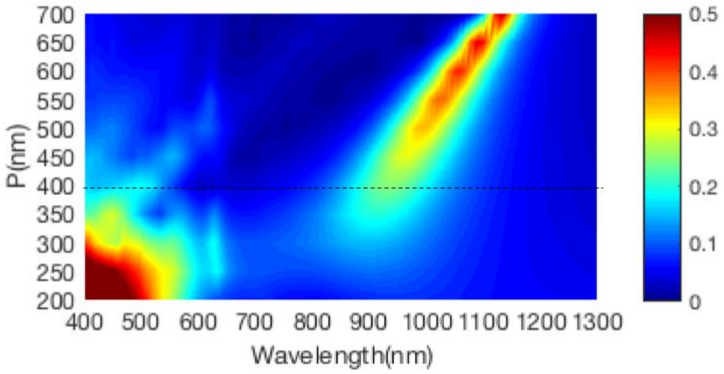

Figure S9. Optical responses of the metasurface composed of only Au layer with various P. Simulated (**a**) transmission and (**b**) absorption spectra of the metasurface composed of only Au layer with various P for front illumination.

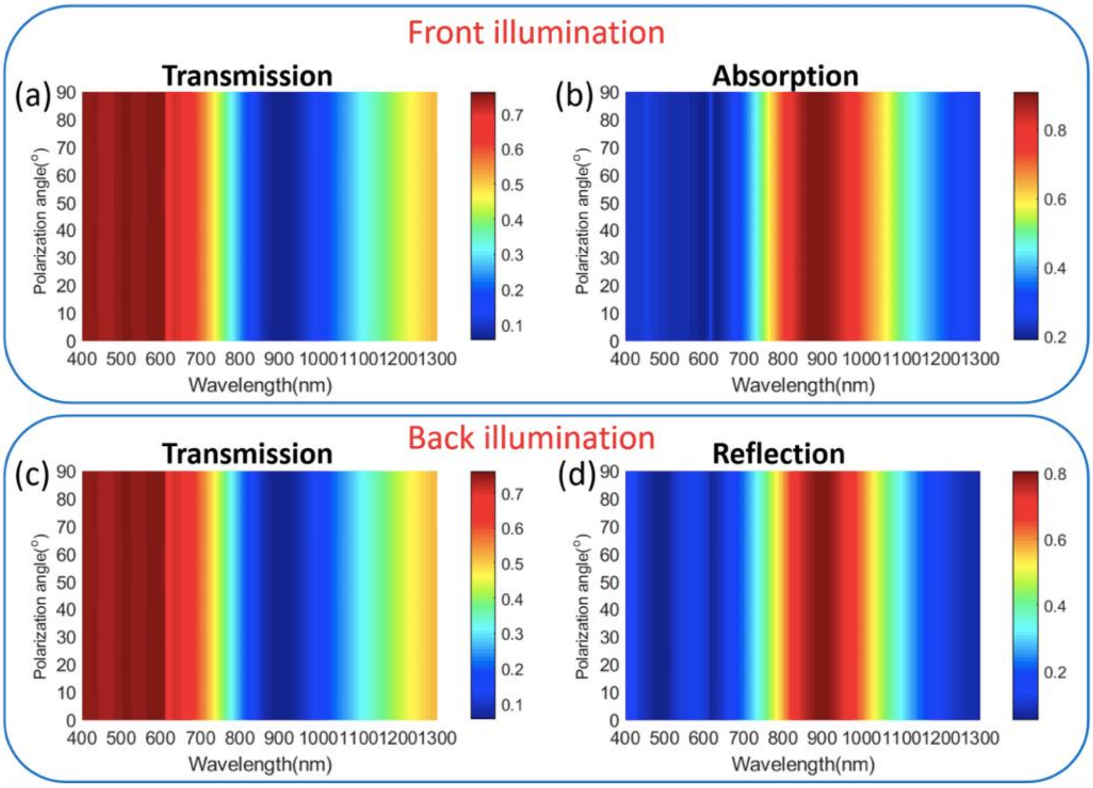

Figure S10. Optical responses of the metasurface consisting of Ti/Al_2O_3/Cu with various polarization angle. Contour plot of the (**a**)transmission and (**b**)absorption of the designed metasurface as a function of wavelength and polarization angle of incident light for front illumination. Contour plot of the (**c**)transmission and (**d**)reflection of the designed metasurface as a function of wavelength and polarization angle of incident light for back illumination.
